# Conditional targeting of MAD1 to kinetochores is sufficient to reactivate the spindle assembly checkpoint in metaphase

**DOI:** 10.1007/s00412-014-0458-9

**Published:** 2014-04-04

**Authors:** Timo E. F. Kuijt, Manja Omerzu, Adrian T. Saurin, Geert J. P. L. Kops

**Affiliations:** 1 Molecular Cancer Research, University Medical Center Utrecht, 3584 CG Utrecht, The Netherlands; 2 Center for Molecular Medicine, University Medical Center Utrecht, 3584 CG Utrecht, The Netherlands; 3 Cancer Genomics Netherlands, University Medical Center Utrecht, 3584 CG Utrecht, The Netherlands; 4 Department of Medical Oncology, Universiteitsweg 100, UMC Utrecht, 3584 CG Utrecht, The Netherlands; 5 Present Address: Medical Research Institute, Jacqui Wood Cancer Centre, University of Dundee, DD1 9SY Dundee, UK

**Keywords:** Spindle checkpoint, Metaphase, MAD1, Kinetochore, Cyclin B1

## Abstract

Fidelity of chromosome segregation is monitored by the spindle assembly checkpoint (SAC). Key components of the SAC include MAD1, MAD2, BUB1, BUB3, BUBR1, and MPS1. These proteins accumulate on kinetochores in early prometaphase but are displaced when chromosomes attach to microtubules and/or biorient on the mitotic spindle. As a result, stable attachment of the final chromosome satisfies the SAC, permitting activation of the anaphase promoting complex/cyclosome (APC/C) and subsequent anaphase onset. SAC satisfaction is reversible, however, as addition of taxol during metaphase stops cyclin B1 degradation by the APC/C. We now show that targeting MAD1 to kinetochores during metaphase is sufficient to reestablish SAC activity after initial silencing. Using rapamycin-induced heterodimerization of FKBP-MAD1 to FRB-MIS12 and live monitoring of cyclin B1 degradation, we show that timed relocalization of MAD1 during metaphase can stop cyclin B1 degradation without affecting chromosome-spindle attachments. APC/C inhibition represented true SAC reactivation, as FKBP-MAD1 required an intact MAD2-interaction motif and MPS1 activity to accomplish this. Our data show that MAD1 kinetochore localization dictates SAC activity and imply that SAC regulatory mechanisms downstream of MAD1 remain functional in metaphase.

**Electronic supplementary material:**

The online version of this article (doi:10.1007/s00412-014-0458-9) contains supplementary material, which is available to authorized users.

## Introduction

Whole chromosome alterations to the karyotype are hazardous to eukaryotic cells (Sheltzer and Amon 2011). As such, a surveillance mechanism named the spindle assembly checkpoint (SAC) has evolved to protect cells from chromosome segregation errors during cell divisions. Our recent comparative genomic analysis showed that this checkpoint was likely present in the last eukaryotic common ancestor, since most protein components of the SAC can be identified in species throughout the eukaryotic tree of life (Vleugel et al. 2012). The SAC monitors the state of attachment of chromosomes to microtubules of the mitotic spindle and halts the cell cycle until all chromosomes have achieved stable biorientation. Unattached kinetochores and/or kinetochores of non-bioriented chromosomes recruit a subset of SAC components that contribute to the generation of a wait-anaphase signal (Musacchio and Salmon 2007; Kops and Shah 2012). Central to this is the MAD1-MAD2 complex that is stably associated with unattached kinetochores (Mapelli and Musacchio 2007). MAD1-MAD2 catalyzes production of an inhibitor of the anaphase-promoting complex/cyclosome (APC/C), resulting in maintenance of sister chromatid cohesion and of the mitotic state (De Antoni et al. 2005; Kulukian et al. 2009; Simonetta et al. 2009). A current model of SAC signaling is as follows: various activities at kinetochores, including BUB1, MPS1, and Rod-ZW10-Zwilch, contribute to recruitment of the MAD1-MAD2 complex (Basto et al. 2000; Brady and Hardwick 2000; Chan et al. 2000; Martin-Lluesma et al. 2002; Meraldi et al. 2004; Kops et al. 2005; Liu et al. 2006; Klebig et al. 2009; Santaguida et al. 2010; Sliedrecht et al. 2010; Maciejowski et al. 2010; Kim et al. 2012; London and Biggins 2014; Moyle et al. 2014). This complex in turn binds soluble MAD2 molecules and converts these into a form that allows association with CDC20, an essential mitotic cofactor of the APC/C (Mapelli and Musacchio 2007). The MAD2-CDC20 complex then binds BUBR1/BUB3 and this four-subunit protein complex, now referred to as the MCC (mitotic checkpoint complex) is directed to the APC/C (Sudakin et al. 2001; Fang 2002; Morrow et al. 2005; Herzog et al. 2009; Tipton et al. 2011; Chao et al. 2012; Tang et al. 2001; Davenport et al. 2006; Kulukian et al. 2009; Elowe et al. 2010; Han et al. 2013). MCC-bound APC/C is incapable of poly-ubiquitinating its metaphase substrates, securin and cyclin B1, at least in large part due to the actions of BUBR1, which occupies a substrate-recognition site on CDC20 and likely has additional inhibitory interactions with the APC/C (Lara-Gonzalez et al. 2011; Chao et al. 2012; Tang et al. 2001; King et al. 2007; Burton and Solomon 2007; Sczaniecka et al. 2008; Pines 2011; Han et al. 2013).

Stable attachment of kinetochores to microtubules causes removal of SAC proteins, thereby negating their ability to generate MCC (Kops and Shah 2012). It was recently shown by Maldonado and Kapoor that removal of MAD1 is a key step in shutting down SAC signaling at kinetochores. Preventing its release after microtubule binding by tethering it to the constitutive kinetochore protein MIS12 delayed anaphase onset (Maldonado and Kapoor 2011). In agreement with this, we showed previously that similar tethering of MPS1 prevented anaphase in human cells and this coincided with persistent MAD1 localization to attached, bioriented kinetochores (Jelluma et al. 2010). While attachment of kinetochores leads to progressive weakening of SAC signaling (Collin et al. 2013), full SAC silencing awaits stable biorientation of all chromosomes. In addition to removal of MAD1 from kinetochores, such silencing requires disassembly of MCC and release of APC/C activity, followed by degradation of cyclin B1 and securin (Reddy et al. 2007; Westhorpe et al. 2011; Varetti et al. 2011; Teichner et al. 2011; Mansfeld et al. 2011; Foster and Morgan 2012; Uzunova et al. 2012). SAC silencing is, however, reversible. Addition of taxol to cells that had initiated cyclin B1 degradation at metaphase was able to rapidly halt further cyclin B1 degradation (Clute and Pines 1999; Dick and Gerlich 2013). Since taxol reduces inter-sister tension and allows a subset of kinetochore-microtubule interactions to be released (Waters et al. 1998), SAC reactivation by taxol in metaphase most likely involved full reactivation of the SAC signaling cascade in response to loss of attachment.

We set out to examine if MAD1 kinetochore-binding is the determining factor in switching the SAC between the ON and OFF state. To this end, MAD1 localization to kinetochores was temporally controlled by chemically induced heterodimerization using the FRB-FKBP12 system (Rivera et al. 1996). Conditional targeting of MAD1 to kinetochores after metaphase and live monitoring of cyclin B1 showed that MAD1 relocalization was sufficient to reactivate the SAC after it was initially silenced.

## Results and discussion

**Fig. 1:**
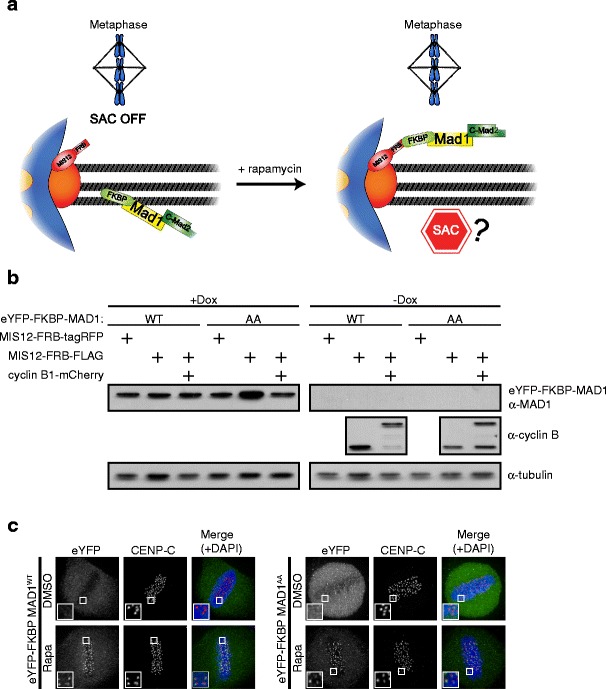
Conditional tethering of MAD1 to kinetochores in human cells. **a** Schematic representation of the experimental system to conditionally relocalize MAD1 to kinetochores. **b** Immunoblots of tubulin, cyclin B1, and eYFP-FKBP-MAD1 (anti-MAD1) from mitotic lysates of various cell lines used in this study. Doxycyline (+Dox) was added 16 h prior to harvesting. **c** Immunostainings of eYFP-FKBP-MAD1 (eYFP, detected with anti-GFP antibody) and kinetochores (CENP-C) in HeLa Flp-in cells expressing MIS12-FRB-FLAG and induced to express eYFP-FKBP-MAD1 (WT, *left* or AA, *right*) by addition of doxycycline for 4 h, and treated with DMSO or rapamycin (rapa) for 30 min in combination with MG132

Constitutive tethering of MAD1 to kinetochores by fusing it to the KMN network component MIS12 prevents SAC silencing in human cells (Maldonado and Kapoor 2011). To examine if MAD1 tethering to kinetochores after SAC silencing is sufficient to reactivate the SAC, we made use of the rapamycin-inducible dimerization of FRB with FKBP12 (Fig. 1a) (Rivera et al. 1996). MIS12 was fused to FRB and stably expressed in HeLa-Flp-in cells that contained a doxycycline-inducible expression cassette for either wild-type (WT) FKBP-MAD1 or a mutant version (K541/L543A) that perturbs MAD2 binding (MAD1^AA^, (Sironi et al. 2002)) (Fig. 1b and S1). MIS12-FRB could be visualized by virtue of a C-terminal FLAG-tagRFP moiety, while the MAD1 proteins could be visualized via an N-terminal eYFP moiety. As expected, 30 min of rapamycin addition to mitotic cells caused accumulation of both FKBP-MAD1 variants on kinetochores (Fig. 1c). In the absence of rapamycin, metaphase kinetochores were devoid of FKBP-MAD1 (Fig. 1c).

**Fig. 2:**
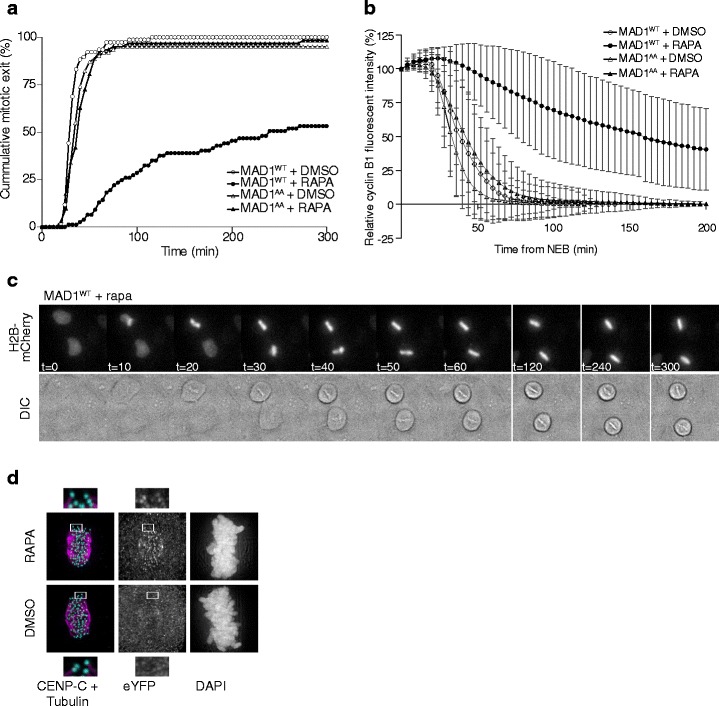
Rapamycin-induced kinetochore tethering of MAD1 prior to mitosis delays SAC silencing. **a** Time-lapse analysis of mitotic progression. HeLa Flp-in cells expressing MIS12-FRB-FLAG were induced to express the FKBP-MAD1 variants by addition of doxycycline 8 h prior to mitotic entry following release from a single thymidine block. DMSO or rapamycin were added 4 h after doxycycline addition. Data (*n* = 50 cells per condition, one representative experiment of three is shown) indicate cumulative fraction of cells that exit from mitosis (as scored by cell morphology using DIC) at the indicated time after NEBD. **b** Time-lapse analysis of cyclin B1 levels during mitotic progression. HeLa Flp-in cells expressing MIS12-FRB-FLAG and cyclin B1-mCherry were induced to express the FKBP-MAD1 variants by addition of doxycycline 8 h prior to mitotic entry following release from a single thymidine block. DMSO or rapamycin was added 4 h after doxycycline addition, after which cells were monitored for cyclin B1-mCherry fluorescence every 5 min. Data (*n* = 40 cells per condition, one representative experiment of two is shown) represent the level of mCherry fluorescence relative to the level at NEBD. **c** Time-lapse analysis of mitotic progression of Flp-in HeLa cells expressing MIS12-FRB-FLAG, induced to express FKBP-MAD1^WT^ by addition of doxycycline for 8 h following release from a single thymidine block and infected with a H2B-mCherry BacMam virus. DMSO or rapamycin were added 4 h after doxycycline addition, after which cells were monitored for morphology (DIC, single plane) and chromosomes (H2B-mCherry, max projection) every 10 min. **d** Immunostainings of cold-stable tubulin, kinetochores (CENP-C) and eYFP-FKBP-MAD1 (eYFP) of HeLa cells expressing MIS12-FRB-FLAG and eYFP-FKBP-MAD1^WT^ and treated with rapamycin (rapa) for 30 min in combination with MG132

To verify that conditional tethering of MAD1 to kinetochores could delay anaphase onset like previously shown for direct fusion of MAD1 to MIS12 (Maldonado and Kapoor 2011), we added rapamycin to a population of cells in G2 phase and monitored mitotic progression by live cell differential interference contrast (DIC) imaging. As shown in Fig. 2a, progression through mitosis was delayed when cells expressing MIS12-FRB and FKBP-MAD1^WT^ were treated with rapamycin. In contrast, mitotic progression occurred with normal timing in the absence of rapamycin (Fig. 2a, DMSO) or when rapamycin was added to cells expressing the FKBP-MAD1^AA^ mutant. These finding were corroborated by monitoring the levels of cyclin B1-mCherry: degradation of cyclin B1 occurred with normal kinetics in the three control conditions but was strongly inhibited in rapamycin-treated cells expressing FKBP-MAD1^WT^ (Fig. 2b). This implied that the observed mitotic delays were due to persistent inhibition of the APC/C by the SAC. SAC activity under these conditions was not due to destabilization of kinetochore-microtubule interactions: rapamycin-treated FKBP-MAD1^WT^ cells were able to rapidly align their chromosomes and remained arrested in mitosis without loss of metaphase plate integrity (Fig. 2c and S2), and the amount and appearance of cold-stable microtubules at metaphase were indistinguishable from control (Fig. 2d).

**Fig. 3:**
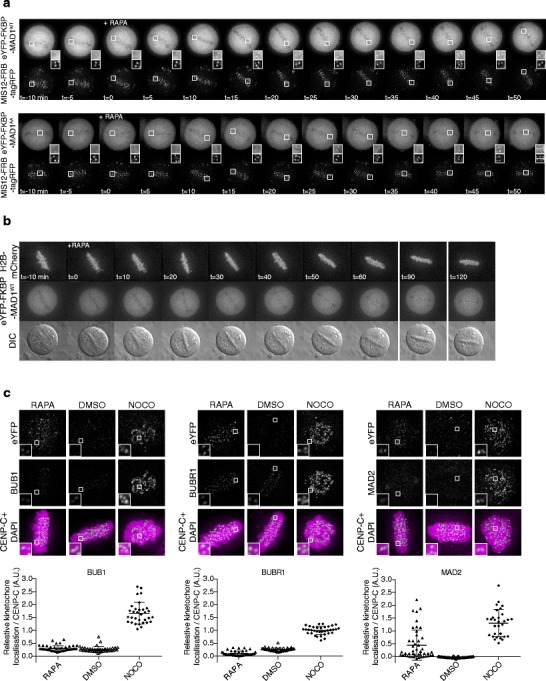
FRB-MAD1 can be recruited to metaphase kinetochores without affecting chromosome alignment. **a**, **b** Time-lapse analysis of MAD1 recruitment (**a**) or chromosome alignment (**b**). HeLa Flp-in cells expressing MIS12-FRB-tagRFP (**a**) or MIS12-FRB-FLAG (**b**) were induced to express the eYFP-FKBP-MAD1 variants by addition of doxycycline for 4 h. MG132 was added for 45 min and metaphase cells were selected for time-lapse imaging. **a** DMSO or rapamycin were added as indicated and cells were imaged every 5 min. **b** HeLa Flp-in cells were infected with H2B-mCherry BacMam virus for 24 h and treated as in (**a**). Metaphase cells were selected and imaged every 10 min. Shown are single plane images of DIC and eYFP and max projections of H2B-mCherry. **c** *Upper panels*: Immunostainings of BUB1 (*left*), BUBR1 (*middle*), and MAD2 (*right*) in combination with kinetochores (CENP-C) and eYFP-FKBP-MAD1 (eYFP) of HeLa Flp-in cells expressing MIS12-FRB-FLAG and induced to express eYFP-FKBP-MAD1^WT^ by addition of doxycycline for 4.5 h. MG132 and DMSO or rapamycin were added for 20 min after cells had reached metaphase, for the duration of 30 minutes. *Lower graphs*: Quantifications of the corresponding immunostainings. Each *dot* represents total kinetochore intensity of a single cell (arbitrary units as a ratio over CENP-C). Averages and standard deviation are indicated.

Thus far, our findings indicate that chemically induced targeting of MAD1 to kinetochores recapitulated published phenotypes of the constitutively kinetochore-tethered MIS12-MAD1 fusion protein (Maldonado and Kapoor 2011). To examine if MAD1 can be recruited to kinetochores in metaphase, we allowed cells to reach metaphase before adding rapamycin. To this end, cells were treated with the proteasome inhibitor MG132 for 30 min after which rapamycin was added for an additional 20 min. Live cell and immunofluorescence imaging showed that MAD1 was efficiently recruited to metaphase kinetochores under these conditions (Fig. 3a–c). Moreover, endogenous MAD2 accumulated on metaphase kinetochores of rapamycin-treated cells expressing FKBP-MAD1^WT^ (Fig. 3c).

To time the speed with which MAD1 could be recruited to kinetochores in metaphase, we followed MG132-treated cells by time-lapse imaging. Kinetochores were monitored by imaging MIS12-FRB-tagRFP and cells were determined to be in metaphase when all kinetochores had aligned on the cell’s equator. Clear MAD1 kinetochore binding could be seen 10–15 min after addition of rapamycin to metaphase cells, as evidenced by accumulation of YFP signals to MIS12-tagRFP-positive kinetochores (Fig. 3a). This timing was comparable for the two MAD1 variants. The induced heterodimerization was relatively slow compared to the speed with which two soluble proteins can be induced to interact, and this may be due to the geometry or microtubule occupancy of the metaphase kinetochore.

Like induced recruitment before mitosis (Figs. 1 and 2), kinetochore recruitment of MAD1 in metaphase did not affect chromosome alignment (Fig. 3b and S3), indicating that conditional targeting of MAD1 in metaphase did not perturb kinetochore-microtubule interactions. In support of this, BUB1 and BUBR1—proteins that accumulate on kinetochores in the absence of interkinetochore tension (Skoufias et al. 2001; Taylor et al. 2001; Ditchfield et al. 2003; Hauf et al. 2003; Howell et al. 2004; Morrow et al. 2005; Famulski and Chan 2007)—were undetectable at metaphase kinetochores to which MAD1 was chemically recruited (Fig. 3c).

Together, these data show that MAD1 can be recruited within 15 min to metaphase kinetochores without affecting chromosome-spindle attachments. This therefore permitted examination of the direct effects of kinetochore MAD1 on SAC activity after metaphase.

**Fig. 4:**
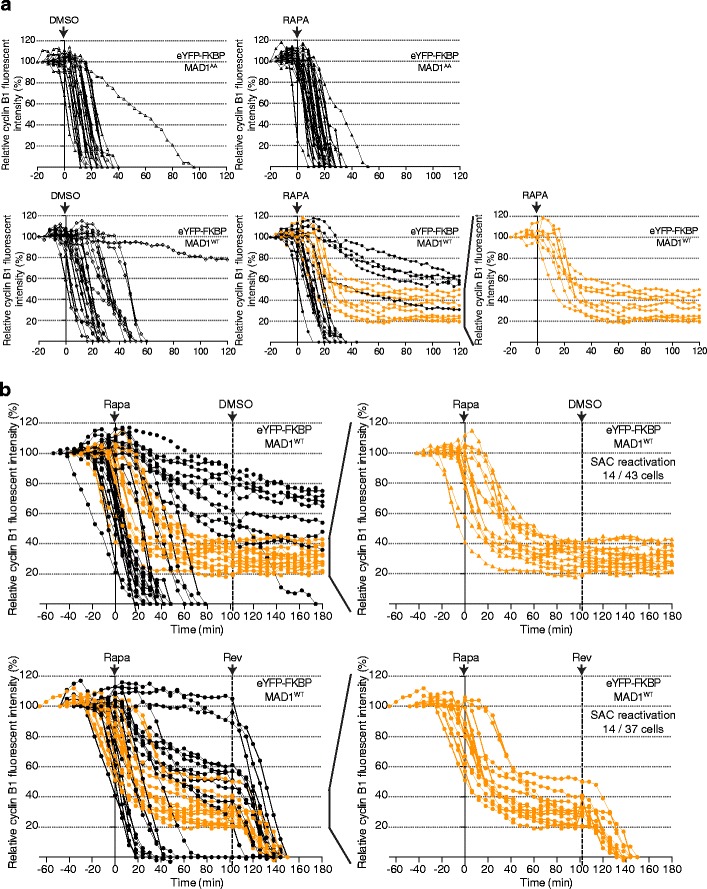
MAD1 recruitment to metaphase kinetochores re-activates the SAC. **a**, **b** Time-lapse analysis of cyclin B1-mCherry. HeLa Flp-in cells expressing MIS12-FRB-FLAG and cyclin B1-mCherry were induced to express the FKBP-MAD1 variants by addition of doxycycline immediately following release from a single thymidine block and imaging started 8 h after that. Rapamycin/DMSO were added during a mitotic wave, after which cells were monitored for cyclin B1-mCherry fluorescence every 5 min. Fluorescent intensity on *y*-axis is relative to intensity at NEBD. **b** 500 nM reversine was added 102 min after rapamycin. **a**, **b** A proportion of cells showed cyclin B1 stabilization after initial decline only in FKBP-MAD1^WT^ cells treated with rapamycin (*orange traces*). Those traces are also separately depicted in *right graphs*. Experiments were performed four (**a**) or two (**b**) times, and one representative experiment is shown in each graph

To be certain that the SAC was silenced by the time we forced MAD1 accumulation on kinetochores, we continuously monitored cyclin B1 levels. Rapamycin was added during the time-lapse experiment at the height of a mitotic wave in the population that occurred roughly 10 hours after release from a thymidine block, when most cells were still in prometaphase. The reasoning was that in at least a fraction of the cells this would allow MAD1 to reach significant levels at kinetochores during degradation of cyclin B1 and before anaphase initiation. In all control situations (FKBP-MAD1^WT^/DMSO, FKBP-MAD1^AA^/DMSO, and FKBP-MAD1^AA^/rapa), cyclin B1 was degraded with comparable kinetics, and anaphase was initiated when most cyclin B1 was degraded (Fig. 4). As expected, however, rapamycin addition to cells expressing FKBP-MAD1^WT^ resulted in three different outcomes. First, cyclin B1 degradation continued as normal, indicating that FKBP-MAD1^WT^ either did not efficiently target to kinetochores in these cells or that it accumulated too late to prevent anaphase onset. Second, cyclin B1 degradation never started, indicating that MAD1 was targeted before the SAC was silenced, similar to rapamycin addition before mitotic entry (Figs. 1 and 2). Third, in roughly one third of the cells (a similar fraction as cells showing significant MAD2 relocalization (Fig. 3c)), cyclin B1 degradation started but was subsequently abrogated (Fig. 4). This behavior was never seen in any of the control situations and showed at single cell level that relocalization of MAD1 to kinetochores was able to reactivate the SAC after it had initially been silenced. This reestablishment of the SAC nevertheless still depended on MPS1 activity as addition of the MPS1 inhibitor reversine (Santaguida et al. 2010) lifted the reinstated block on cyclin B1 degradation and caused cells to initiate anaphase (Fig. 4b and S4) without affecting kinetochore FKBP-MAD1 levels (Fig. S4). It may be of interest to note that all cells that restabilized cyclin B1 after rapamycin addition did so with at least 20 % of cyclin B1 left. This may indicate that in this cell line, a significant amount of cyclin B1 is needed to either maintain the mitotic state and/or support SAC reactivation, in agreement with recent reports (Dick and Gerlich 2013; Vázquez-Novelle et al. 2014).

Our data show that forced localization of MAD1 to metaphase kinetochores is sufficient to reactivate functional SAC signaling after initial silencing. This implies that MAD1 removal is a key step in SAC silencing. Inhibition of pathways that recruit MAD1 (e.g., MPS1, RZZ, BUB1) combined with activation of pathways that displace MAD1 (e.g., dynein, spindly, kinetochore phosphatases (Wojcik et al. 2001; Howell et al. 2001; Yang et al. 2007; Pinsky et al. 2009; Vanoosthuyse and Hardwick 2009; Gassmann et al. 2010; Barisic et al. 2010; Famulski et al. 2011; Rosenberg et al. 2011)) will thus be required to maintain the silenced state until anaphase. Key unresolved issues are the nature and spatiotemporal regulation of these pathways and their relation to kinetochore-microtubule interactions. An intriguing player in this is MPS1. Persistent MPS1 localization to metaphase kinetochores causes persistent MAD1 kinetochore binding (Jelluma et al. 2010), so MPS1 itself needs to be removed from kinetochores at metaphase to allow MAD1 removal and SAC silencing. At the same time, MPS1 remains active and able to contribute to SAC signaling, since SAC reactivation by conditional MAD1 tethering can be reverted by the MPS1 inhibitor reversine (Fig. 4b). This implies that at least part of the SAC signaling pathways that contribute downstream of (or in parallel to) MAD1 kinetochore binding are still operational at metaphase. How some aspects of MPS1 function are maintained so as to assure SAC reactivation if required but some are repressed so as to allow MAD1 removal is an interesting challenge for further research.

## Methods

### Cell culture and reagents

HeLa Flp-in cells (gift from S. Taylor, University of Manchester, England, UK) stably expressing a TetR, were cultured in DMEM (4.5 g/L glucose, Lonza) supplemented with 9 % fetal bovine serum (Tetracyclin-approved, Lonza), 50 μg/ml penicillin/streptomycin (Gibco), and 2 mM Ultraglutamine (Lonza). All HeLa Flp-in cell lines stably carrying doxycycline-inducible eYFP-FKBP-MAD1 constructs were transfected with pcDNA5/FRT/TO (Invitrogen) and pOG44 (Invitrogen) plasmid-carrying Flp-recombinase. Selection and maintenance of stable cells was done in medium supplemented with 200 μg/ml Hygromycin B (Roche) and 4 μg/ml blasticidin (PAA Laboratories). HeLa Flp-in cell lines stably expressing MIS12-FRB constructs were transfected with Fugene HD (Roche), and stable lines were selected for using 2 μg/ml puromycin (Sigma). The HeLa Flp-in cell lines expressing cyclin B1-mCherry were transfected with pcDNA3-cyclin B1-mCherry and, stable cell lines were selected using 100 μg/ml Zeocin (Invivogen). The reagents thymidine (2 mM), reversine (500 nM), nocodazole (830 μM), MG132 (10 μM), and doxycycline (1 μg/ml) were purchased from Sigma-Aldrich and used at final concentrations indicated. Rapamycin (100 nM) was purchased from LC-Laboratories.

### Plasmids

To create pcDNA5-eYFP-FKBP-MAD1^WT^ and -MAD1^AA^ constructs, FKBP12 (a gift from Lukas Kapitein) was PCR-amplified, ligated into pcDNA5-LAP-MAD1 using HindIII sites, and the sequence was verified. MIS12-FRB-tagRFP (MIS12-FRB-FLAG-tagRFP-IRES-PURO) and MIS12-FRB-FLAG (MIS12-FRB-FLAG-IRES-PURO) were constructed as follows: FRB was amplified from GFP-FRB (Gift of Klaus Hahn) and inserted into pc3-FLAG-tagRFP using EcoRI/ClaI sites to create pc3-FRB-FLAG-tagRFP. MIS12 was amplified from pcDNA3-MIS12-MPS1 (Jelluma et al. 2010) and inserted (AscI/NheI) into pIRES-PURO (a gift of Susanne Lens). FRB-FLAG-tagRFP was then amplified from pc3-FRB-FLAG-tagRFP and inserted (NheI/NotI) into pMIS12-IRES-PURO. pcDNA3-cyclin B1-mCherry plasmid was created by inserting a HindIII-NotI fragment of pcDNA5-cyclin B1-mCherry into pcDNA3. The neomycin selection gene of pcDNA3 was subsequently replaced with Zeocin using NotI/MluI.

### Immunofluorescence

HeLa Flp-in cells were plated on 12-mm round coverslips (No. 1.5) and induction of eYFP-FKBP-MAD1 was done for 4.5 h. Cells were pre-extracted using 37 °C PEMT (100 mM PIPES (pH 6.8), 1 mM MgCl2, 5 mM EGTA, 0.2 % Triton X-100) for 1 min after which cells were fixed in 4 % paraformaldehyde/PBS for 15 min. Coverslips were blocked in 3 % BSA/PBS for 1 h and primary antibody incubations were done overnight at 4 °C. Coverslips were washed three times in PBS/0.1 % TX-100 and subsequently incubated with secondary antibodies plus DAPI for 1 h at room temperature. Coverslips were washed twice in PBS and mounted using ProLong Gold antifade (Molecular Probes). Image acquisition was done on a DeltaVision RT system (Applied Precision/GE Healthcare) with a 100 × 1.40 numerical aperture (NA) UPlanSApo objective (Olympus) and for deconvolution SoftWorx (Applied Precision/GE Healthcare) was used. Image analysis and quantification was done using ImageJ and image preparation for figures was done using Photoshop and Illustrator CS5 (Adobe Systems). All graphs were created in Graphpad Prism 6.0d (GraphPad Software, La Jolla, CA, USA).

The following primary antibodies were used for immunofluorescence imaging: GFP (custom rabbit polyclonal, 1:10.000), GFP (Abcam, mouse monocolonal 1:1,000), BUB1 (Bethyl, A300-373A, 1:1,000), BUBR1 (Bethyl, A300-386A, 1:1,000), MAD2 (custom rabbit polyclonal antibody, 1:1,000), and CENP-C (MBL Life Science, polyclonal Guinea pig, PD030, 1:2,000). Secondary antibodies used for immunofluorescence were highly crossed absorbed anti-guinea pig Alexa Fluor 647, anti-rabbit and anti-mouse Alexa Fluor 488, and 568, anti-rat Alexa Fluor 568 (Molecular Probes).

### Live-cell imaging

Differential interference contrast (DIC) microscopy was performed on an Olympus IX81 inverted microscope equipped with a 10 × 0.30 NA CPlanFLN objective lens (Olympus), Hamamatsu ORCA-ER camera and Cell^M software (Olympus). Time-lapse imaging of cells plated in a 12-well plate, was done at 37 °C and 5 % CO_2_ concentration. Images were acquired every 5 min at 2 × 2 binning and analysis of time-lapse movies was done using ImageJ software where the time between nuclear envelope breakdown (NEBD) and anaphase-onset was determined.

For live-cell fluorescent imaging of cyclin B1-mCherry degradation above described system was used. Imaged were acquired every 5 min 1 × 1 binning (1,024 × 1,024 pixels). Sample illumination was kept to a minimum to prevent perturbing cell viability.

Live-cell imaging of eYFP-FKBP-MAD1 was performed on a personal DeltaVision system (Applied Precision/GE Healthcare) equipped with a Coolsnap HQ2 CCD camera (Photometrics) and Insight solid-state illumination (Applied Precision/GE Healthcare). Images were acquired every 5 min using a 100 × 1.4 NA UPlanSApo objective (Olympus) at 2× 2 binning. Twelve-micrometer-thick optical sections were acquired at 4 μm steps and YFP illumination was set to 100 ms and 50 % neutral density (ND) filter, mCherry illumination was set to 150 ms and 50 % ND. For H2B-mCherry, live-cell imaging the mCherry illumination was set to 50 ms and 50 % ND. Images were deconvolved using standard settings in SoftWorx (Applied Precision/GE Healthcare). For imaging analysis, Image J was used and figure preparation was done in Illustrator CS5 (Adobe).

### Immunoblotting

Cells were blocked in thymidine for 20 h and released for 16 h in presence of nocodazole and doxycycline when indicated. Mitotic cells were collected by shake-off and cells were lysed in 2× Laemmli sample buffer. Cell lysates were boiled for 5 min and separated on a 10 % SDS-PAGE gel. Proteins were transferred to nitrocellulose membranes and membranes were blocked in 5 % milk/TBS-0.1 % Tween-20 for 30 min. The following primary antibodies were used: anti-tubulin (clone B-5-1-2; Sigma; T5168, 1:10.000), anti-MAD1 (Fig. 1b and S1B: M-300; Santa Cruz; sc-67337 1:1,000; Fig. S1A: Sigma M-8069, 1:1,000) and anti-cyclin B1 (GNS1; Santa Cruz; sc-245, 1:1,000). Detection of proteins was done with HRP-conjugated secondary antibodies (Bio-Rad) and chemiluminescence. Adobe Photoshop and Illustrator were used to create the figure.

## Electronic supplementary material

Below is the link to the electronic supplementary material.

Supplemental Figure 1 Immuneblots of lysates from various FKBP-MAD1 cell lines. A) Long-exposure immuoblot of eYFP-FKBP-MAD1 (anti-MAD1, Sigma M-8069) from mitotic lysates of indicated cell lines. B) Full size immunoblots of experiment in 1B. Marks indicate crop lines used in Figure 1B. Endogenous MAD1 is not visible because of high overexpression of exogenous MAD1 and short exposure time. (PDF 192 kb)

Supplemental Figure 2 Rapamycin-induced tethering of MAD1^AA^ prior to mitosis. Time-lapse analysis of mitotic progression of Flp-in HeLa cells expressing MIS12-FRB-FLAG, induced to express FKBP-MAD1^AA^ and infected with a H2B-mCherry BacMam virus 24 hours. Cells were treated as described in Figure 2A. Depicted are single plane DIC and max projection stills of H2B-mCherry. (PDF 93 kb)

Supplemental Figure 3 FRB-MAD1 can be recruited to metaphase kinetochores without affecting chromosome alignment. A) Stills from time-lapse analysis of chromosome alignment in FKBP-MAD1^WT^ cells. Cells were treated as described in Figure 3B. B) Immunostainings of BUB1 in combination with kinetochores (CENP-C) and eYFP-FKBP-MAD1 (eYFP) of HeLa Flp-in cells expressing MIS12-FRB-FLAG and induced to express eYFP-FKBP-MAD1^WT^ by addition of doxycycline for four and a half hours. MG132 for 30 minutes and the inhibitors were added for 20 minutes after cells had reached metaphase. Addition of DMSO or rapamycin in combination with reversine was done simultaneously for 20 minutes. Graph indicates quantification of the corresponding immunostaining. Each dot represents total kinetochore intensity of a single cell (arbitrary units as a ratio over CENP-C). Averages and standard deviation are indicated. (PDF 1125 kb)

Supplemental Figure 4 MAD1 recruitment to metaphase kinetochores re-activates the SAC in an MPS1-dependent manner. A) Stills from time-lapse analysis of HeLa Flp-in cells expressing cyclin B1-mCherry and induced to express eYFP-FKBP-MAD1^WT^. Experimental conditions are as described for Figure 4B. Indicated are the time of rapamycin addition (t = 0 min) and time of 500 nM reversine addition (t = 102 min). B) Stills of time-lapse analysis of FKBP-MAD1 during metaphase arrest before and after reversine addition. Cells were treated exactly as in Figure 4b. (PDF 754 kb)
