# Supplementary figures and images for: Conditional targeting of MAD1 to kinetochores is sufficient to reactivate the spindle assembly checkpoint in metaphase
Source: Chromosoma. 2014 Apr 4;123(5):471–80. doi: 10.1007/s00412-014-0458-9 (PMC4169584; doi:10.1007/s00412-014-0458-9)

**a**

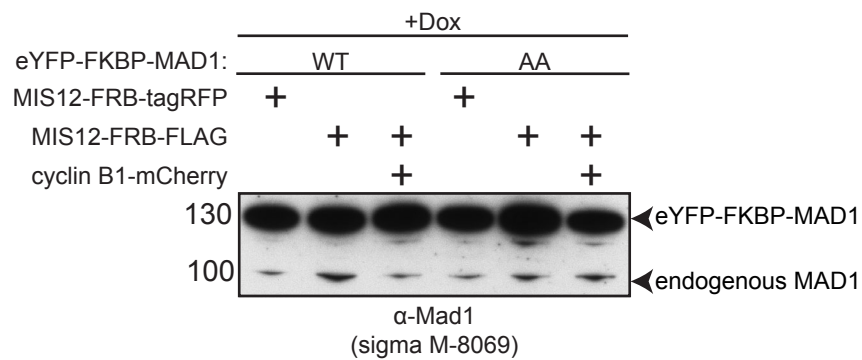

**b**

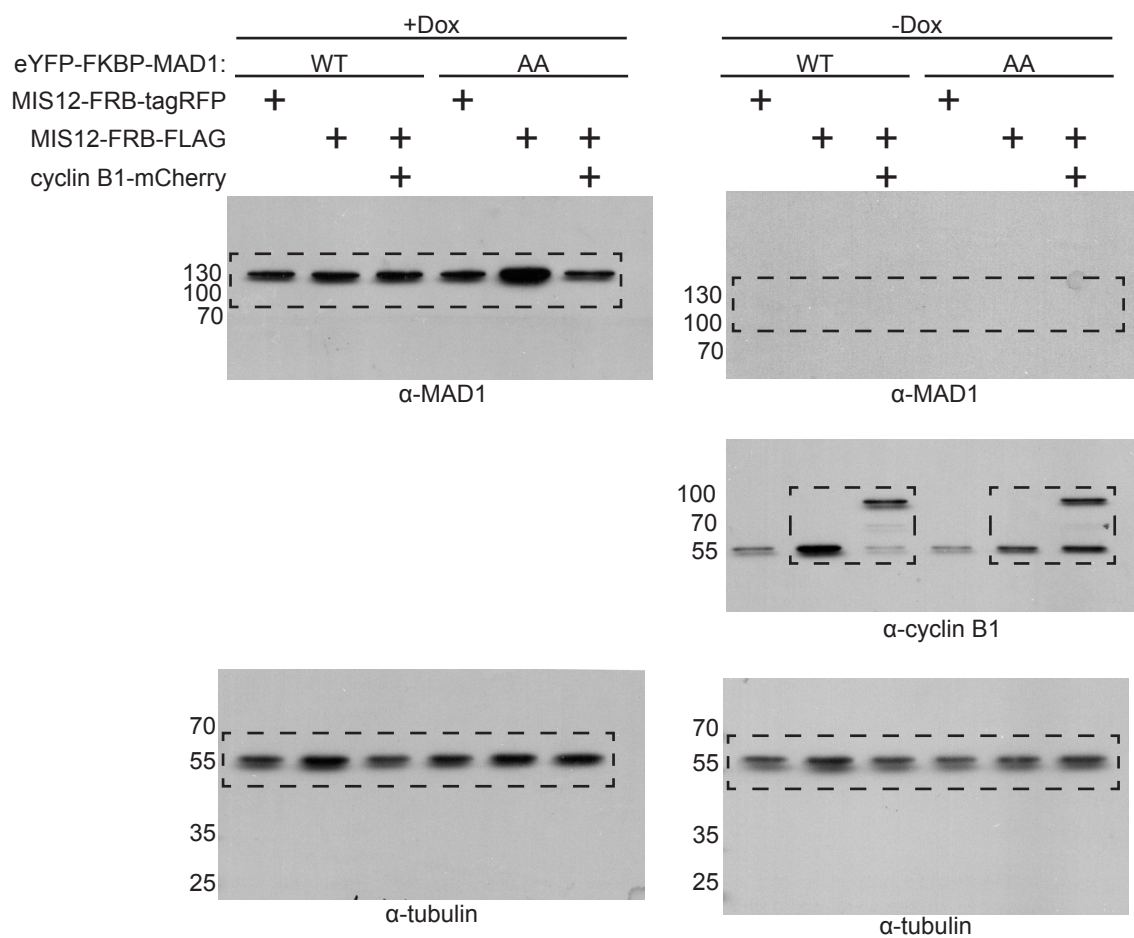

Supplement: Supplementary file 1 — Immuneblots of lysates from various FKBP-MAD1 cell lines. A) Long-exposure immuoblot of eYFP-FKBP-MAD1 (anti-MAD1, Sigma M-8069) from mitotic lysates of indicated cell lines. B) Full size immunoblots of experiment in 1B. Marks indicate crop lines used in Figure 1B. Endogenous MAD1 is not visible because of high overexpression of exogenous MAD1 and short exposure time. (PDF 192 kb) [file 412_2014_458_MOESM1_ESM.pdf]

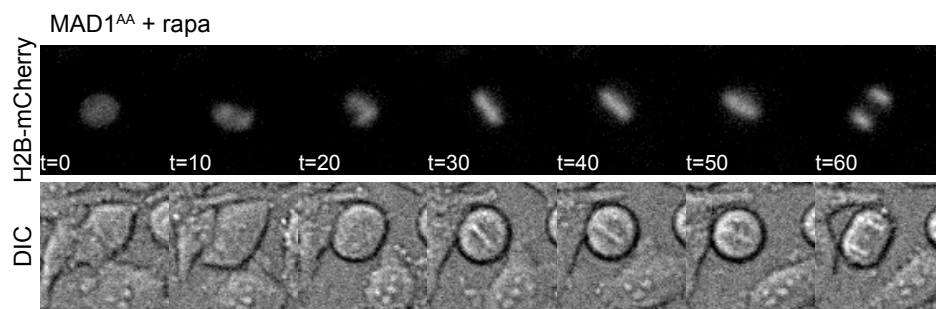

Kuijt et al., Supplemental Figure 2

Supplement: Supplementary file 2 — Rapamycin-induced tethering of MAD1AA prior to mitosis. Time-lapse analysis of mitotic progression of Flp-in HeLa cells expressing MIS12-FRB-FLAG, induced to express FKBP-MAD1AA and infected with a H2B-mCherry BacMam virus 24 hours. Cells were treated as described in Figure 2A. Depicted are single plane DIC and max projection stills of H2B-mCherry. (PDF 93 kb) [file 412_2014_458_MOESM2_ESM.pdf]

**a**

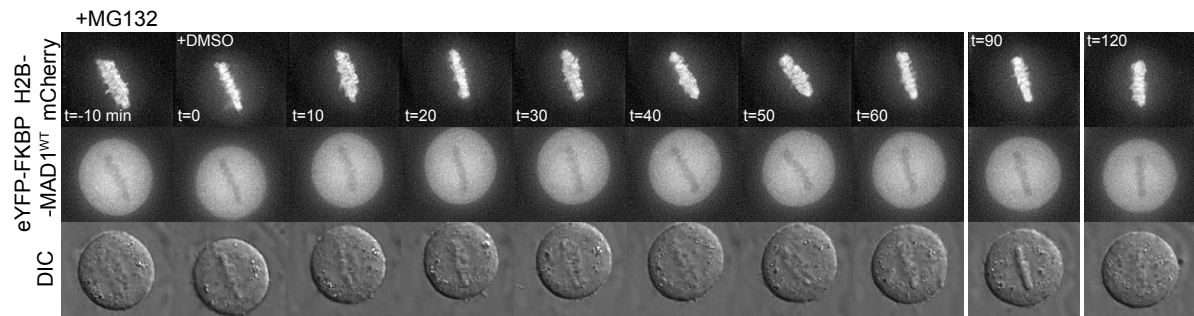

**b**

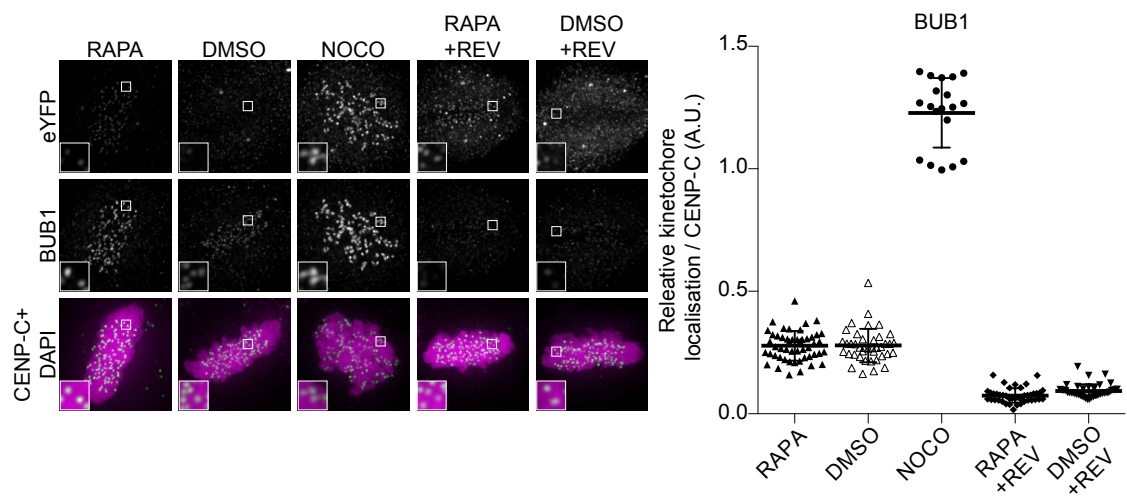

Kuijt et al., Supplemental Figure 3

Supplement: Supplementary file 3 — FRB-MAD1 can be recruited to metaphase kinetochores without affecting chromosome alignment. A) Stills from time-lapse analysis of chromosome alignment in FKBP-MAD1WT cells. Cells were treated as described in Figure 3B. B) Immunostainings of BUB1 in combination with kinetochores (CENP-C) and eYFP-FKBP-MAD1 (eYFP) of HeLa Flp-in cells expressing MIS12-FRB-FLAG and induced to express eYFP-FKBP-MAD1WT by addition of doxycycline for four and a half hours. MG132 for 30 minutes and the inhibitors were added for 20 minutes after cells had reached metaphase. Addition of DMSO or rapamycin in combination with reversine was done simultaneously for 20 minutes. Graph indicates quantification of the corresponding immunostaining. Each dot represents total kinetochore intensity of a single cell (arbitrary units as a ratio over CENP-C). Averages and standard deviation are indicated. (PDF 1125 kb) [file 412_2014_458_MOESM3_ESM.pdf]

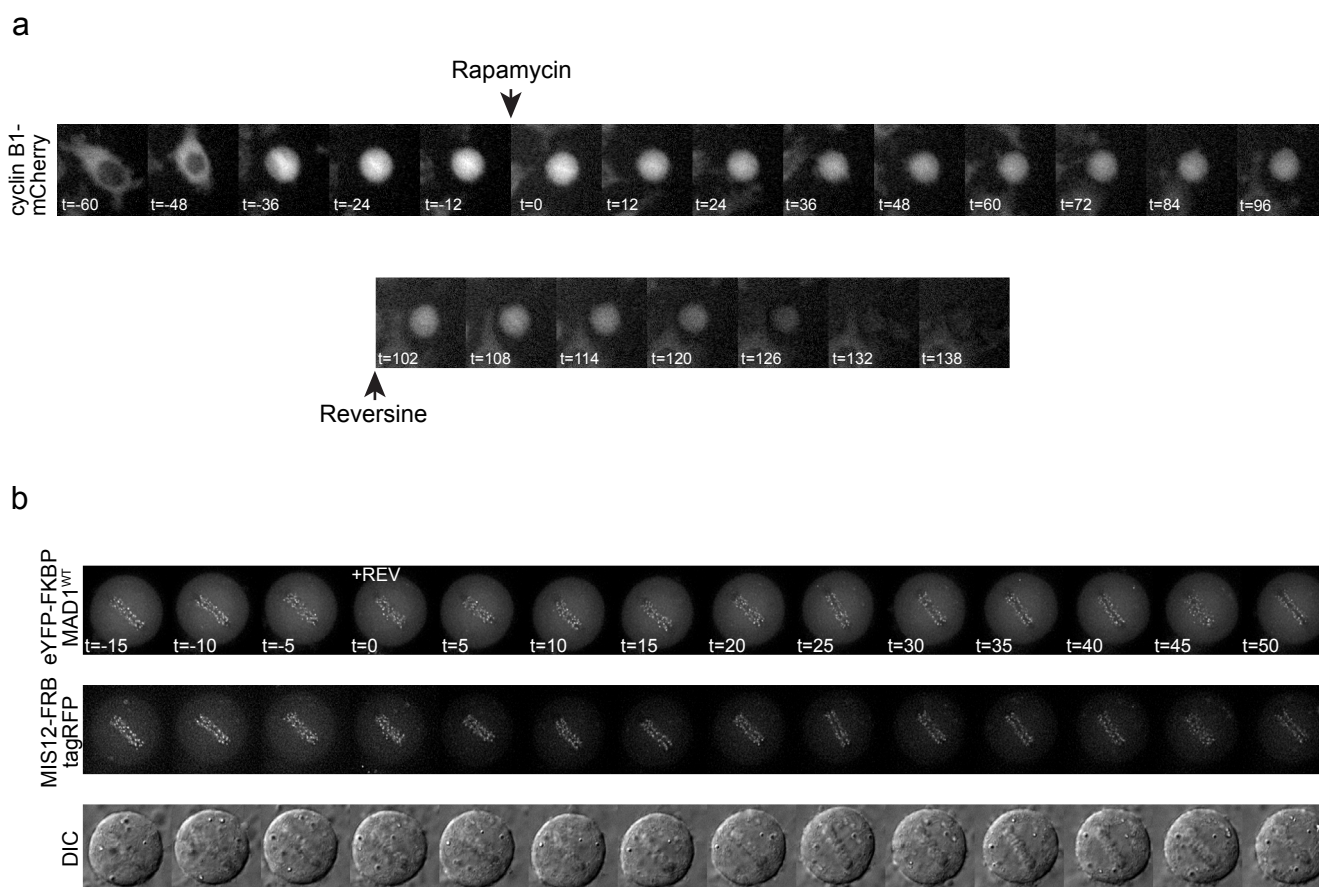

Kuijt et al., Supplemental Figure 4

Supplement: Supplementary file 4 — MAD1 recruitment to metaphase kinetochores re-activates the SAC in an MPS1-dependent manner. A) Stills from time-lapse analysis of HeLa Flp-in cells expressing cyclin B1-mCherry and induced to express eYFP-FKBP-MAD1WT. Experimental conditions are as described for Figure 4B. Indicated are the time of rapamycin addition (t = 0 min) and time of 500 nM reversine addition (t = 102 min). B) Stills of time-lapse analysis of FKBP-MAD1 during metaphase arrest before and after reversine addition. Cells were treated exactly as in Figure 4b. (PDF 754 kb) [file 412_2014_458_MOESM4_ESM.pdf]
